# Diaqua­bis­(2,2′-bi-1*H*-imidazole)­man­ganese(II) benzene-1,4-di­carboxyl­ate

**DOI:** 10.1107/S160053681202199X

**Published:** 2012-05-26

**Authors:** Lining Yang, Yanxiang Zhi, Jiahui Hei, Yanqing Miao

**Affiliations:** a Department of Pharmacy, Xi’an Medical University, Xi’an, Shaanxi 710021, People’s Republic of China; b Department of Chemistry, Northwest University, Xi’an, Shaanxi 710069, People’s Republic of China

## Abstract

The asymmetric unit of the title compound, [Mn(C_6_H_6_N_4_)_2_(H_2_O)_2_](C_8_H_4_O_4_), contains one-half each of the centrosymmetric cation and anion. The Mn^II^ atom is coordinated by four N atoms [Mn—N = 2.2168 (14) and 2.2407 (14) Å] from two 2,2′-biimidazole ligands and two water mol­ecules [Mn—O = 2.2521 (14) Å] in a distorted octa­hedral geometry. Inter­molecular N—H⋯O and O—H⋯O hydrogen bonds consol­idate the crystal packing, which also exhibits π–π inter­actions between five-membered rings, with a centroid–centroid distance of 3.409 (2) Å.

## Related literature

For related structures, see: Fortin & Beauchamp (2001 ▶); Sang *et al.* (2002 ▶); Atencio *et al.* (2004 ▶); Wang *et al.* (2007 ▶). For background to supra­molecular assemblies, see: Ramirez *et al.* (2002 ▶); Baca *et al.* (2003 ▶).
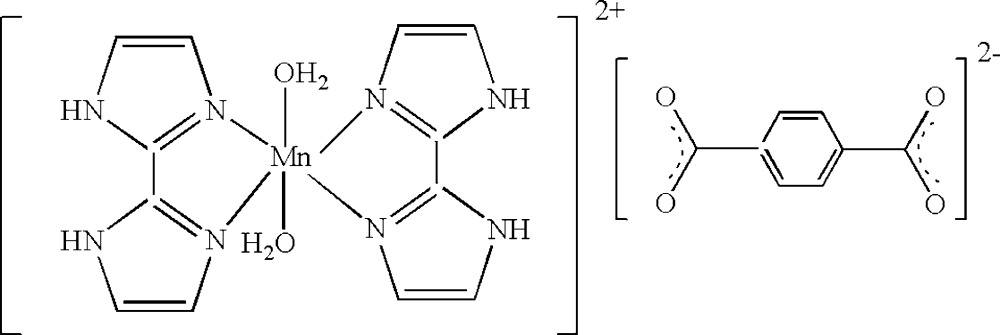


## Experimental

### 

#### Crystal data

[Mn(C_6_H_6_N_4_)_2_(H_2_O)_2_](C_8_H_4_O_4_)
*M* *_r_* = 523.38
Monoclinic, 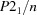

*a* = 8.2666 (10) Å
*b* = 10.9027 (13) Å
*c* = 12.6734 (16) Å
β = 93.986 (2)°
*V* = 1139.5 (2) Å^3^
*Z* = 2
Mo *K*α radiation
μ = 0.63 mm^−1^
*T* = 293 K
0.46 × 0.19 × 0.07 mm

#### Data collection

Bruker SMART CCD area-detector diffractometer
Absorption correction: multi-scan (*SADABS*; Bruker, 2004 ▶) *T*_min_ = 0.761, *T*_max_ = 0.960
5689 measured reflections
2024 independent reflections
1712 reflections with *I* > 2σ(*I*)
*R*_int_ = 0.022

#### Refinement

*R*[*F* ^2^ > 2σ(*F* ^2^)] = 0.027
*wR*(*F* ^2^) = 0.075
*S* = 1.04
2024 reflections
167 parameters
H atoms treated by a mixture of independent and constrained refinement
Δρ_max_ = 0.20 e Å^−3^
Δρ_min_ = −0.17 e Å^−3^

### 

Data collection: *SMART* (Bruker, 2004 ▶); cell refinement: *SAINT* (Bruker, 2004 ▶); data reduction: *SAINT*; program(s) used to solve structure: *SHELXS97* (Sheldrick, 2008 ▶); program(s) used to refine structure: *SHELXL97* (Sheldrick, 2008 ▶); molecular graphics: *SHELXTL* (Sheldrick, 2008 ▶); software used to prepare material for publication: *SHELXL97*.

## Supplementary Material

Crystal structure: contains datablock(s) I, global. DOI: 10.1107/S160053681202199X/cv5293sup1.cif

Structure factors: contains datablock(s) I. DOI: 10.1107/S160053681202199X/cv5293Isup2.hkl

Additional supplementary materials:  crystallographic information; 3D view; checkCIF report

## Figures and Tables

**Table 1:** Hydrogen-bond geometry (Å, °)

*D*—H⋯*A*	*D*—H	H⋯*A*	*D*⋯*A*	*D*—H⋯*A*
O3—H3*B*⋯O1^i^	0.80 (3)	2.09 (3)	2.850 (2)	159 (3)
O3—H3*A*⋯O2^ii^	0.87 (3)	1.85 (3)	2.711 (2)	170 (3)
N4—H4⋯O1	0.86	1.87	2.7101 (19)	165
N2—H2*A*⋯O2	0.86	1.89	2.7482 (19)	173

Symmetry codes: (i) 
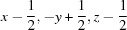
; (ii) 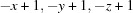.

**Fig. 1.**
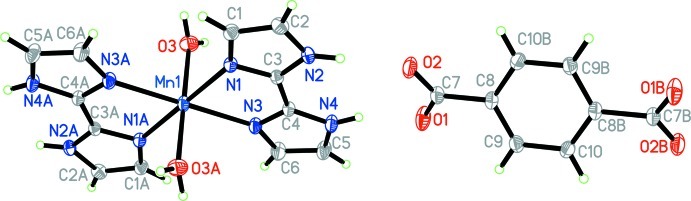
View of the title complex with the atom-numbering scheme [symmetry codes: (A) -x, -y + 1, -z + 1; (B) -x + 2, -y + 1, -z + 2]. Displacement ellipsoids are drawn at the 30% probability level.

## supplementary crystallographic information

### Comment

Supramolecular assemblies have provided numerous materials with very attractive properties (Ramirez *et al.*, 2002). One of the best strategies to construct such materials relies on the use of both the building block approach and the additional hydrogen bonding of the coordinated ligands to their linking capability (Baca *et al.*, 2003). The 2,2'-biimidazole (H_2_biim) possesses these properties - coordination to metal centre and acting as a donor in hydrogen bonding interaction (Fortin & Beauchamp, 2001; Sang *et al.*, 2002; Atencio *et al.*, 2004; Wang *et al.*, 2007).

The structure of the title complex consists of [Mn(H_2_biim)_2_(H_2_O)_2_]^2+^ cations and terephthalate dianions (Fig. 1). The coordination geometry of the manganese(II) centre can be described as a distorted octahedron including four nitrogen atoms from two chelating H_2_biim ligands and two oxygen atoms from aqua ligands. The crystal packing is stabilized by the hydrogen bonds N—H···O and O—H···O (Table 1).

### Experimental

All reagents were of AR grade from commercial sources and used without further purification. Biimidazole was prepared following the known procedure (Ramirez *et al.*, 2002). Mn(CH_3_COO)_2_ (0.3 mmol), H_2_biim (0.3 mmol) and terephthalic acid (0.3 mmol) in the molar ratio of 1:1:1 were added directly as a solid in 10 ml deionized water respectively, after the mixture was stirred at room temperature for 30 min, the pH value was adjusted to 7.0 by aqueous KOH solution. Then the mixture was placed in a 25 ml Teflon-lined stainless steel vessel and heated at 160°C for 6 days under autogenous pressure. Afterwards, the vessel was cooled to room temperature at a rate of 10°C per hour. Light yellow sheet-like crystals of title complex were obtained and collected by filtration and washed with water (yield 40%).

### Refinement

The O-bound H atoms were located in difference Fourier maps and were refined with restraints O—H=0.84 (3) Å and U_iso_(H) fixed to 0.08 Å^2^. The rest H atoms were geometrically positioned [C—H = 0.93 Å; N—H = 0.86 Å], and treated as riding, with *U*_iso_(H) = 1.2*U*_eq_ of the parent atom.

### Crystal data

**Table:**

[Mn(C_6_H_6_N_4_)_2_(H_2_O)_2_](C_8_H_4_O_4_)	*F*(000) = 538
*M**_r_* = 523.38	*D*_x_ = 1.525 Mg m^−^^3^
Monoclinic, *P*2_1_/*n*	Mo *K*α radiation, λ = 0.71073 Å
*a* = 8.2666 (10) Å	Cell parameters from 2178 reflections
*b* = 10.9027 (13) Å	θ = 3.0–26.9°
*c* = 12.6734 (16) Å	µ = 0.63 mm^−^^1^
β = 93.986 (2)°	*T* = 293 K
*V* = 1139.5 (2) Å^3^	Block, yellow
*Z* = 2	0.46 × 0.19 × 0.07 mm

### Data collection

**Table:**

Bruker SMART CCD area-detector diffractometer	2024 independent reflections
Radiation source: fine-focus sealed tube	1712 reflections with *I* > 2σ(*I*)
Graphite monochromator	*R*_int_ = 0.022
φ and ω scans	θ_max_ = 25.1°, θ_min_ = 2.5°
Absorption correction: multi-scan (*SADABS*; Bruker, 2004)	*h* = −6→9
*T*_min_ = 0.761, *T*_max_ = 0.960	*k* = −13→12
5689 measured reflections	*l* = −14→15

### Refinement

**Table:**

Refinement on *F*^2^	Secondary atom site location: difference Fourier map
Least-squares matrix: full	Hydrogen site location: inferred from neighbouring sites
*R*[*F*^2^ > 2σ(*F*^2^)] = 0.027	H atoms treated by a mixture of independent and constrained refinement
*wR*(*F*^2^) = 0.075	*w* = 1/[σ^2^(*F*_o_^2^) + (0.0403*P*)^2^ + 0.2933*P*] where *P* = (*F*_o_^2^ + 2*F*_c_^2^)/3
*S* = 1.04	(Δ/σ)_max_ < 0.001
2024 reflections	Δρ_max_ = 0.20 e Å^−^^3^
167 parameters	Δρ_min_ = −0.17 e Å^−^^3^
0 restraints	Extinction correction: *SHELXL97* (Sheldrick, 2008), Fc^*^=kFc[1+0.001xFc^2^λ^3^/sin(2θ)]^-1/4^
Primary atom site location: structure-invariant direct methods	Extinction coefficient: 0.0048 (12)

### Special details

**Table:**

Geometry. All e.s.d.'s (except the e.s.d. in the dihedral angle between two l.s. planes) are estimated using the full covariance matrix. The cell e.s.d.'s are taken into account individually in the estimation of e.s.d.'s in distances, angles and torsion angles; correlations between e.s.d.'s in cell parameters are only used when they are defined by crystal symmetry. An approximate (isotropic) treatment of cell e.s.d.'s is used for estimating e.s.d.'s involving l.s. planes.
Refinement. Refinement of *F*^2^ against ALL reflections. The weighted *R*-factor *wR* and goodness of fit *S* are based on *F*^2^, conventional *R*-factors *R* are based on *F*, with *F* set to zero for negative *F*^2^. The threshold expression of *F*^2^ > σ(*F*^2^) is used only for calculating *R*-factors(gt) *etc*. and is not relevant to the choice of reflections for refinement. *R*-factors based on *F*^2^ are statistically about twice as large as those based on *F*, and *R*- factors based on ALL data will be even larger.

### Fractional atomic coordinates and isotropic or equivalent isotropic displacement parameters (Å^2^)

**Table:**

	*x*	*y*	*z*	*U*_iso_*/*U*_eq_	
Mn1	0.0000	0.5000	0.5000	0.02879 (15)	
N1	0.24692 (16)	0.58566 (12)	0.51483 (10)	0.0273 (3)	
N2	0.48208 (17)	0.58683 (13)	0.60838 (11)	0.0289 (3)	
H2A	0.5580	0.5673	0.6552	0.035*	
N3	0.12437 (17)	0.39115 (14)	0.62924 (11)	0.0316 (4)	
N4	0.34624 (18)	0.37195 (14)	0.73441 (11)	0.0354 (4)	
H4	0.4422	0.3851	0.7628	0.042*	
O1	0.62726 (17)	0.39434 (14)	0.85633 (11)	0.0517 (4)	
O2	0.72528 (16)	0.54524 (15)	0.76328 (11)	0.0501 (4)	
O3	0.08176 (19)	0.36279 (14)	0.38200 (11)	0.0431 (4)	
C1	0.3431 (2)	0.67589 (16)	0.47712 (14)	0.0322 (4)	
H1	0.3136	0.7280	0.4210	0.039*	
C2	0.4879 (2)	0.67729 (16)	0.53438 (14)	0.0335 (4)	
H2	0.5744	0.7298	0.5250	0.040*	
C3	0.3359 (2)	0.53425 (15)	0.59437 (13)	0.0248 (4)	
C4	0.2721 (2)	0.43418 (15)	0.65354 (12)	0.0272 (4)	
C5	0.2410 (2)	0.28425 (19)	0.76286 (16)	0.0432 (5)	
H5	0.2592	0.2268	0.8166	0.052*	
C6	0.1051 (2)	0.29636 (18)	0.69827 (15)	0.0403 (5)	
H6	0.0128	0.2479	0.7003	0.048*	
C7	0.7311 (2)	0.47488 (17)	0.84132 (14)	0.0342 (4)	
C8	0.8711 (2)	0.48881 (15)	0.92349 (14)	0.0297 (4)	
C9	0.8507 (2)	0.45947 (17)	1.02814 (14)	0.0337 (4)	
H9	0.7504	0.4323	1.0476	0.040*	
C10	0.9791 (2)	0.47042 (17)	1.10393 (14)	0.0345 (4)	
H10	0.9643	0.4502	1.1739	0.041*	
H3A	0.135 (3)	0.390 (3)	0.330 (2)	0.080*	
H3B	0.104 (3)	0.292 (3)	0.391 (2)	0.080*	

### Atomic displacement parameters (Å^2^)

**Table:**

	*U*^11^	*U*^22^	*U*^33^	*U*^12^	*U*^13^	*U*^23^
Mn1	0.0234 (2)	0.0332 (2)	0.0286 (2)	−0.00110 (16)	−0.00624 (14)	0.00314 (16)
N1	0.0261 (8)	0.0281 (8)	0.0272 (7)	0.0002 (6)	−0.0017 (6)	0.0020 (6)
N2	0.0229 (8)	0.0310 (8)	0.0321 (8)	−0.0009 (6)	−0.0041 (6)	−0.0006 (6)
N3	0.0259 (8)	0.0362 (9)	0.0316 (8)	−0.0039 (7)	−0.0047 (6)	0.0066 (6)
N4	0.0290 (8)	0.0414 (9)	0.0342 (8)	−0.0042 (7)	−0.0085 (6)	0.0097 (7)
O1	0.0465 (9)	0.0514 (9)	0.0536 (9)	−0.0173 (7)	−0.0224 (7)	0.0128 (7)
O2	0.0357 (8)	0.0730 (10)	0.0397 (8)	−0.0108 (7)	−0.0124 (6)	0.0198 (7)
O3	0.0497 (9)	0.0394 (8)	0.0410 (8)	−0.0008 (7)	0.0093 (6)	−0.0038 (7)
C1	0.0342 (10)	0.0297 (10)	0.0327 (9)	0.0005 (8)	0.0015 (8)	0.0055 (8)
C2	0.0306 (10)	0.0300 (10)	0.0404 (10)	−0.0061 (8)	0.0045 (8)	0.0008 (8)
C3	0.0229 (9)	0.0249 (9)	0.0261 (8)	0.0011 (7)	−0.0007 (7)	−0.0022 (7)
C4	0.0260 (9)	0.0291 (9)	0.0261 (9)	0.0005 (7)	−0.0026 (7)	0.0014 (7)
C5	0.0409 (11)	0.0451 (12)	0.0426 (11)	−0.0073 (9)	−0.0056 (9)	0.0216 (9)
C6	0.0327 (10)	0.0434 (12)	0.0439 (11)	−0.0111 (9)	−0.0035 (8)	0.0144 (9)
C7	0.0289 (10)	0.0397 (11)	0.0331 (10)	−0.0006 (8)	−0.0055 (8)	−0.0006 (8)
C8	0.0299 (10)	0.0273 (9)	0.0308 (9)	0.0027 (7)	−0.0066 (7)	−0.0007 (7)
C9	0.0268 (10)	0.0384 (10)	0.0354 (10)	−0.0015 (8)	−0.0021 (7)	0.0008 (8)
C10	0.0354 (11)	0.0398 (11)	0.0274 (9)	−0.0008 (8)	−0.0026 (8)	0.0010 (8)

### Geometric parameters (Å, º)

**Table:**

Mn1—N3	2.2168 (14)	O2—C7	1.250 (2)
Mn1—N3^i^	2.2168 (14)	O3—H3A	0.87 (3)
Mn1—N1	2.2407 (14)	O3—H3B	0.80 (3)
Mn1—N1^i^	2.2407 (14)	C1—C2	1.356 (2)
Mn1—O3	2.2521 (14)	C1—H1	0.9300
Mn1—O3^i^	2.2521 (14)	C2—H2	0.9300
N1—C3	1.330 (2)	C3—C4	1.444 (2)
N1—C1	1.372 (2)	C5—C6	1.350 (3)
N2—C3	1.338 (2)	C5—H5	0.9300
N2—C2	1.364 (2)	C6—H6	0.9300
N2—H2A	0.8600	C7—C8	1.510 (2)
N3—C4	1.324 (2)	C8—C10^ii^	1.383 (3)
N3—C6	1.370 (2)	C8—C9	1.386 (3)
N4—C4	1.341 (2)	C9—C10	1.386 (2)
N4—C5	1.358 (2)	C9—H9	0.9300
N4—H4	0.8600	C10—C8^ii^	1.383 (3)
O1—C7	1.252 (2)	C10—H10	0.9300
			
N3—Mn1—N3^i^	180.0	C2—C1—N1	109.46 (15)
N3—Mn1—N1	77.77 (5)	C2—C1—H1	125.3
N3^i^—Mn1—N1	102.23 (5)	N1—C1—H1	125.3
N3—Mn1—N1^i^	102.23 (5)	C1—C2—N2	106.73 (15)
N3^i^—Mn1—N1^i^	77.77 (5)	C1—C2—H2	126.6
N1—Mn1—N1^i^	180.00 (6)	N2—C2—H2	126.6
N3—Mn1—O3	89.42 (6)	N1—C3—N2	111.53 (15)
N3^i^—Mn1—O3	90.58 (6)	N1—C3—C4	120.62 (15)
N1—Mn1—O3	91.08 (5)	N2—C3—C4	127.86 (15)
N1^i^—Mn1—O3	88.92 (5)	N3—C4—N4	111.31 (15)
N3—Mn1—O3^i^	90.58 (6)	N3—C4—C3	120.73 (15)
N3^i^—Mn1—O3^i^	89.42 (6)	N4—C4—C3	127.95 (15)
N1—Mn1—O3^i^	88.92 (5)	C6—C5—N4	106.95 (16)
N1^i^—Mn1—O3^i^	91.08 (5)	C6—C5—H5	126.5
O3—Mn1—O3^i^	180.00 (5)	N4—C5—H5	126.5
C3—N1—C1	105.24 (14)	C5—C6—N3	109.40 (16)
C3—N1—Mn1	109.91 (11)	C5—C6—H6	125.3
C1—N1—Mn1	144.74 (11)	N3—C6—H6	125.3
C3—N2—C2	107.05 (14)	O2—C7—O1	124.18 (17)
C3—N2—H2A	126.5	O2—C7—C8	118.01 (17)
C2—N2—H2A	126.5	O1—C7—C8	117.81 (16)
C4—N3—C6	105.36 (14)	C10^ii^—C8—C9	119.03 (16)
C4—N3—Mn1	110.79 (11)	C10^ii^—C8—C7	121.06 (16)
C6—N3—Mn1	143.79 (12)	C9—C8—C7	119.91 (16)
C4—N4—C5	106.97 (15)	C8—C9—C10	120.33 (17)
C4—N4—H4	126.5	C8—C9—H9	119.8
C5—N4—H4	126.5	C10—C9—H9	119.8
Mn1—O3—H3A	117.7 (18)	C8^ii^—C10—C9	120.64 (17)
Mn1—O3—H3B	129 (2)	C8^ii^—C10—H10	119.7
H3A—O3—H3B	108 (3)	C9—C10—H10	119.7
			
N3—Mn1—N1—C3	−3.35 (11)	C1—N1—C3—C4	179.99 (15)
N3^i^—Mn1—N1—C3	176.65 (11)	Mn1—N1—C3—C4	2.77 (19)
N1^i^—Mn1—N1—C3	118.74 (12)	C2—N2—C3—N1	0.25 (19)
O3—Mn1—N1—C3	−92.52 (11)	C2—N2—C3—C4	−179.90 (17)
O3^i^—Mn1—N1—C3	87.48 (11)	C6—N3—C4—N4	−0.3 (2)
N3—Mn1—N1—C1	−178.7 (2)	Mn1—N3—C4—N4	177.63 (11)
N3^i^—Mn1—N1—C1	1.3 (2)	C6—N3—C4—C3	178.54 (16)
N1^i^—Mn1—N1—C1	−56.59 (18)	Mn1—N3—C4—C3	−3.5 (2)
O3—Mn1—N1—C1	92.1 (2)	C5—N4—C4—N3	0.4 (2)
O3^i^—Mn1—N1—C1	−87.9 (2)	C5—N4—C4—C3	−178.38 (18)
N3^i^—Mn1—N3—C4	−180 (81)	N1—C3—C4—N3	0.5 (3)
N1—Mn1—N3—C4	3.61 (11)	N2—C3—C4—N3	−179.33 (16)
N1^i^—Mn1—N3—C4	−176.39 (11)	N1—C3—C4—N4	179.15 (16)
O3—Mn1—N3—C4	94.84 (12)	N2—C3—C4—N4	−0.7 (3)
O3^i^—Mn1—N3—C4	−85.16 (12)	C4—N4—C5—C6	−0.3 (2)
N3^i^—Mn1—N3—C6	−3 (81)	N4—C5—C6—N3	0.1 (2)
N1—Mn1—N3—C6	−179.8 (2)	C4—N3—C6—C5	0.1 (2)
N1^i^—Mn1—N3—C6	0.2 (2)	Mn1—N3—C6—C5	−176.60 (16)
O3—Mn1—N3—C6	−88.5 (2)	O2—C7—C8—C10^ii^	−30.4 (3)
O3^i^—Mn1—N3—C6	91.5 (2)	O1—C7—C8—C10^ii^	150.38 (18)
C3—N1—C1—C2	0.00 (19)	O2—C7—C8—C9	150.21 (18)
Mn1—N1—C1—C2	175.45 (14)	O1—C7—C8—C9	−29.0 (3)
N1—C1—C2—N2	0.1 (2)	C10^ii^—C8—C9—C10	−0.3 (3)
C3—N2—C2—C1	−0.24 (19)	C7—C8—C9—C10	179.15 (17)
C1—N1—C3—N2	−0.15 (18)	C8—C9—C10—C8^ii^	0.3 (3)
Mn1—N1—C3—N2	−177.37 (11)		

Symmetry codes: (i) −*x*, −*y*+1, −*z*+1; (ii) −*x*+2, −*y*+1, −*z*+2.

### Hydrogen-bond geometry (Å, º)

**Table:**

*D*—H···*A*	*D*—H	H···*A*	*D*···*A*	*D*—H···*A*
O3—H3*B*···O1^iii^	0.80 (3)	2.09 (3)	2.850 (2)	159 (3)
O3—H3*A*···O2^iv^	0.87 (3)	1.85 (3)	2.711 (2)	170 (3)
N4—H4···O1	0.86	1.87	2.7101 (19)	165
N2—H2*A*···O2	0.86	1.89	2.7482 (19)	173

Symmetry codes: (iii) *x*−1/2, −*y*+1/2, *z*−1/2; (iv) −*x*+1, −*y*+1, −*z*+1.
